# An exploratory fetal MRI study examining the impact of 22q11.2 microdeletion syndrome on early brain growth

**DOI:** 10.1186/s11689-025-09594-9

**Published:** 2025-02-12

**Authors:** Daniel Cromb, Tom Finck, Alexandra F. Bonthrone, Alena Uus, Milou Van Poppel, Johannes Steinweg, David F Lloyd, Kuberan Pushparajah, Reza Razavi, Serena J. Counsell, Mary Rutherford

**Affiliations:** 1 Research Department of Early Life Imaging, Department of Perinatal Imaging and Health, School of Biomedical Engineering and Imaging Sciences, Faculty of Life Sciences and Medicine, King’s College London, London, UK; 2 Department of Diagnostic and Interventional Neuroradiology, Klinikum rechts der Isar, Technischen Universität München, Munich, Germany; 3 Department of Cardiovascular Imaging, School of Biomedical Engineering & Imaging Science, King’s College London, London, UK; 4 Biomedical Engineering Department, School of Biomedical Engineering and Imaging Sciences, King’s College London, London, UK; 5 MRC Centre for Neurodevelopmental Disorders, King’s College London, London, UK

**Keywords:** 22q deletion syndrome, Fetal MRI

## Abstract

**Background:**

Improved long-term outcomes, related to advances in surgical and clinical care of infants with congenital heart disease (CHD), has shifted focus onto the accompanying and later-onset cognitive and neuropsychiatric disorders in those who also have 22q11.2 deletion syndrome (22qDS). 22qDS is itself associated with neurodevelopmental impairments and altered brain growth. However, when brain growth in 22qDS first deviates from normal is unknown, and whether impaired brain development is primarily genetics-driven or a secondary consequence of the underlying CHD remains incompletely understood.

**Methods:**

In this small, exploratory study, we use fetal MRI to assess volumetric brain development in 22qDS by comparing fetal brain morphometry to a set of gestation and sex-matched healthy controls, and a cohort of gestation and sex-matched fetuses with the same CHD diagnoses but without 22q11.2 deletion. Structural T2-weighted fetal brain images were acquired using a 1.5T MRI scanner. MR scanner and sequence parameters were identical in all cohorts. Motion-corrected images underwent segmentation using an automated pipeline developed for fetal brain MRI. Total brain tissue volumes, volumes for four different tissue regions (cortical grey matter, white matter, deep grey matter and cerebellum), cerebrospinal fluid and total intracranial volumes were calculated.

**Results:**

Antenatal imaging was acquired between 29 and 35 weeks gestation. Thirty-three fetuses were included (7 22qDS; 14 isolated CHD; 12 healthy control). White matter volumes were significantly reduced in fetuses with 22qDS compared to control fetuses (*p* = 0.028), but not to those with CHD without 22q11.2 deletion (*p* = 0.09). Large effect-sizes were seen between the 22qDS and isolated CHD cohorts (D_Cohen_ = 0.81), and between the 22qDS and control cohorts (D_Cohen_ = 1.2) for white matter volumes. No significant differences were seen in volumes of other brain regions between groups.

**Conclusions:**

This exploratory study expands our existing knowledge on neurodevelopmental impairments in 22qDS to the fetal period by highlighting reduced white matter volumes compared to gestation and sex-matched control fetuses during this time-period. Our findings suggest that impaired white matter growth in fetuses with both 22qDS and CHD may not be fully explained by any underlying CHD.

## Background

DiGeorge syndrome, velocardiofacial syndrome and conotruncal anomaly syndrome are common phenotypes of the 22q11.2 microdeletion spectrum (22qDS). Initially believed to constitute separate diagnoses, they are now grouped together as a spectrum with common aetiology and variable phenotypes [1].

Incidences of 22qDS have been reported at rates of approximately one in a thousand in low-risk pregnancies [2]. Phenotypes, even within the same family, can be heterogeneous depending on the length of missing base pairs, although typical manifestations include congenital heart defects (CHD), palatal abnormalities, immune deficiencies related to thymus hypoplasia, and atypical facial appearances [3, 4]. The associated neurodevelopmental and neuropsychiatric disorders often contribute to an individual’s ability to live an independent life [5, 6] and identifying underlying biological mechanisms associated with these may enable improved counselling and prognoses.

Forging a better understanding of the neuropathophysiology in 22qDS has become a growing area of interest. Studies have highlighted reduced overall brain sizes, along with topographical volume changes in both grey and white matter [7–9] in children and adults with 22qDS. Additionally, more complex neurostructural changes, such as alterations in cortical thickness and/or folding have been suggested as likely mechanisms mediating cognitive impairments [10]. White matter (WM) microstructural alterations have also been reported [11]. Since isolated structural brain findings in 22qDS are rare and generally overshadowed by the clinically more well-described CHD, this renders assumptions on causative effects along the heart-brain axis difficult. As CHD itself is associated with altered brain development, including reduced brain volumes [12–15], the question of whether brain development in 22qDS is defined by the underlying genetics or related to the cardiac phenotype remains not fully understood, although there is reasonable evidence from preclinical models and large-scale genomic studies for a role of specific genes in the locus (e.g. CRKL, TBX1) on cardiac phenotypes [16, 17]. As metabolic demands of the fetal brain are greatest in the third trimester [18], one could hypothesise that structural differences resulting from reduced cerebral oxygenation and substrate delivery in CHD also contribute and might only become apparent at later stages in fetal or neonatal development, while genetically mediated brain growth restriction may present earlier in development [19]. Postnatal imaging may fail to discern timing and may be complicated by peripartum injuries. Assessment is further hampered by the confounder that many children with 22qDS undergo cardiac surgery in the early postnatal period, with potential for further acquired injury, representing additional risk factors for impaired development [20].

MRI is a safe, non-invasive tool suitable for evaluating morphology in the fetal brain and can help with diagnosis of syndromic or prenatally acquired conditions [21, 22]. Improvements in image acquisition and post-processing, with a special focus on the reduction of motion artefacts, continuously increase its clinical value in antenatal diagnostics. Importantly, morphometric assessments allow us to assess trajectories of regional brain growth. Thorough analysis of these trajectories, especially in complex syndromes, may allow clarification on the specific aetiology of atypical brain development.

## Aims

We aim to perform a small exploratory analysis into early structural brain development in 22qDS by comparing fetal brain morphometry to a set of healthy controls, addressing the question of whether impaired brain growth in 22qDS begins in fetal life. In addition, we compared brain findings in 22qDS to a cohort of fetuses with matched CHD diagnoses but without 22q11.2 deletion.

## Methods

### Ethical approval

The National Research Ethics Service West London committee provided ethical approval (22qDS and CHD fetuses: 07/H0707/105; Control fetuses: 14/LO/1806). Informed, written consent was obtained from all participants before undergoing fetal MRI.

### Subjects

For this retrospective study, relevant clinical data from all women referred to the fetal cardiology service at St. Thomas’ Hospital in London who had a fetus with a confirmed or suspected diagnosis of congenital heart disease, undergoing fetal MRI, and who consented to the data being used for research purposes were analysed. Fetuses with 22q11.2 deletion confirmed on antenatal and/or postnatal genetic testing, either via amniocentesis or comparative genomic hybridization array (CGH-array) were identified, forming a ‘22q cohort’.

Subsequently, any fetuses where the antenatal diagnosis of isolated congenital heart disease, the gestational age (GA) at the time of the scan (± 1 week) and the fetal sex matched each of those in the 22q cohort were identified, to establish a ‘matched cardiac’ cohort. Fetal CHD diagnosis for both the 22q and matched cardiac cohorts was made on fetal echocardiography, with all diagnoses being confirmed by a fetal cardiologist.

Women experiencing low-risk pregnancies, undergoing fetal MRI as part of the Intelligent Fetal Imaging and Diagnosis (iFIND) project (https://www.ifindproject.com/) and who consented to the images being used for research were also identified. Fetuses with a matched GA at the time of the scan (± 1 week) and the same fetal sex for each of those in the 22q cohort were identified to establish a ‘matched control’ cohort.

For all cohorts, maternal exclusion criteria included multiple pregnancy, weight over 125 kg, severe claustrophobia, maternal type 1 or 2 diabetes mellitus or gestational diabetes, and chronic or gestational hypertension.

### MR Imaging

#### Image acquisition

Fetal images were acquired on a Philips Ingenia 1.5T MRI scanner, with 28-channel dStream anterior and posterior in-built coils. Maternal comfort was maximised by elevating the head and legs, and continuous monitoring of vital signs with verbal interactions were performed throughout.

Fetal brain images were acquired using a T2-weighted single-shot fast-spin-echo sequence optimised for fetal imaging (Repetition Time [TR] = 15,000msec, Echo Time [TE] = 80msec, voxel size = 1.25 × 1.25 mm, slice thickness = 2.5 mm, slice spacing = 1.25 mm, field-of-view = 420 × 420 mm, flip angle 90°). To image the fetal body, sagittal whole-uterus images were acquired using a balanced steady-state free precession (bSSFP) sequence (TR = 5msec, TE = 1.98msec, voxel size = 1.25 × 1.25 mm, slice thickness = 5 mm, field-of-view = 420 × 420 mm, flip angle = 90°). No sedation or intravenous contrast was used.

#### Fetal brain image reconstruction

**Fig. 1:**
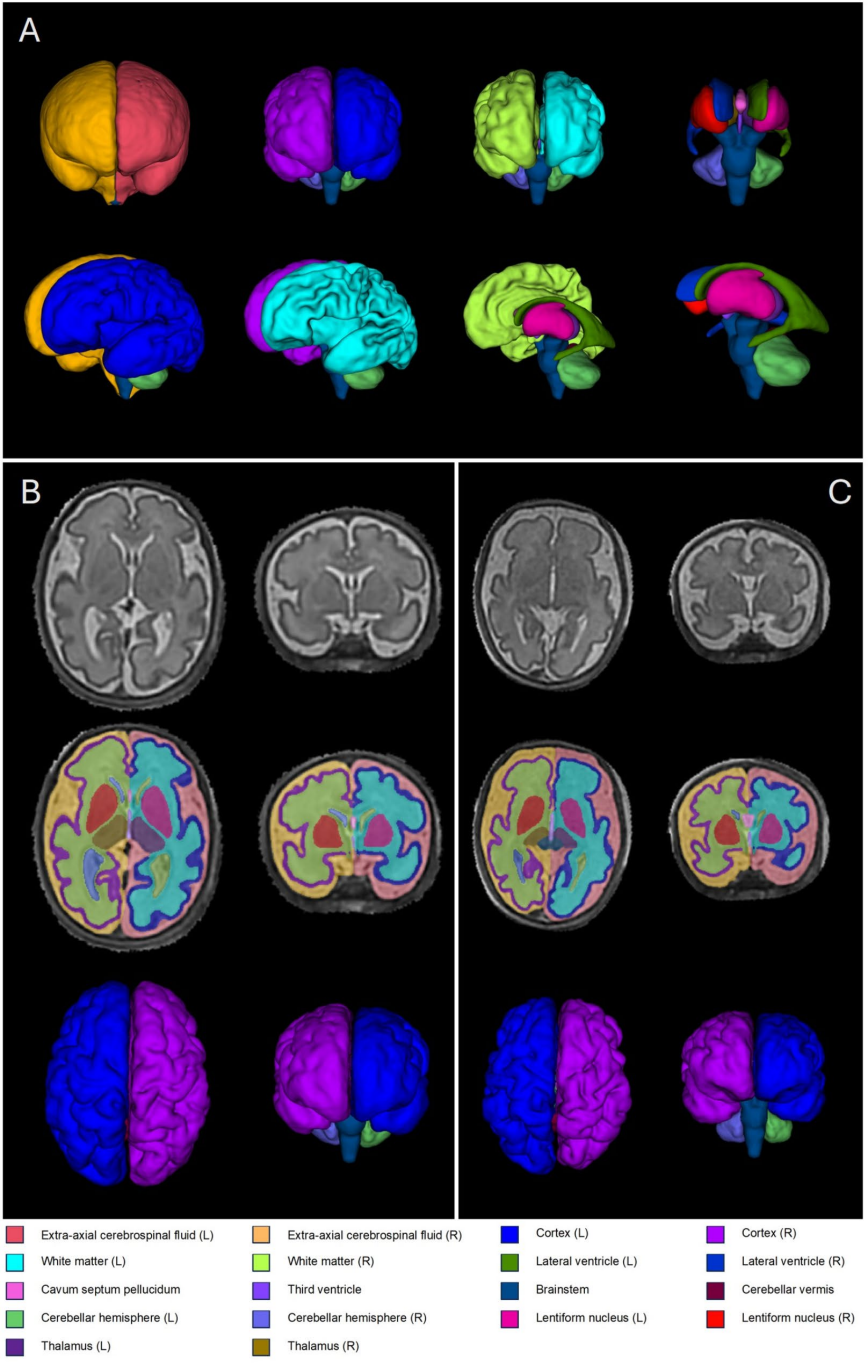
Example 3D volumetric reconstructions of extra-axial cerebrospinal fluid (CSF), cortical grey matter, white matter, deep grey matter structures, internal CSF filled spaces, the cerebellum and the brainstem derived from an automated fetal brain tissue segmentation pipeline (**A**). Examples of good quality 3D fetal brain SVRs in axial and coronal views for a healthy control fetus (**B**) and a fetus with 22qDS (**C**) along with corresponding regional segmentations are also shown

The fetal brain images were motion corrected and reconstructed using an automated version of the classical 3D slice-to-volume registration (SVR) reconstruction technique [23, 24], before being up-sampled to 0.5mm^3^ isotropic resolution (Fig. 1).

#### Image review

All MR images were reported by experienced perinatal neuroradiologists. The presence of any structural abnormalities was recorded. The quality of the fetal brain reconstructions, taking into account the definition of anatomical features, signal to noise ratio, contrast and alignment, was independently assessed by two clinicians trained in the assessment of fetal and neonatal brain MRI, with each image being graded as good (4/4), acceptable (3/4), poor (2/4) or failed (1/4), as previously described [25]. Only datasets where both reviewers gave a rating of acceptable or good were included.

### Fetal brain volumetry

Each brain SVR was segmented using an automated Brain vOlumetry and aUtomated parcellatioN for 3D feTal MRI (BOUNTI) pipeline [26] (SVRTK segmentation docker: https://hub.docker.com/r/fetalsvrtk/ segmentation tag bounti_brain_tissue_1.00) based on the developing Human Connectome Project atlas segmentation protocol with 19 label regions of interest (ROIs) (https://gin.g-node.org/kcl_cdb/fetal_ brain_mri_atlas). All resulting segmentations were reviewed and confirmed to be acceptable for volumetry. No manual editing was performed on segmentations.

The volume of each of the 19 labels was calculated using the FSL utilities *fslmaths* command [27], enabling volumes for multiple brain regions to be calculated: Total brain tissue volume (TTV), cortical grey matter (cGM) volume, white matter (WM) volume, deep grey matter (dGM) volume, cerebellar volume and cerebrospinal fluid (CSF) volume. TTV was calculated by summing cGM, WM, dGM, cerebellar and brainstem volumes. dGM volume was calculated by summing left and right lentiform nuclei and left and right thalamic volumes. Cerebellar volume was calculated by summing left and right cerebellar hemisphere and cerebellar vermis volumes. CSF volume was calculated by summing extra-axial CSF, lateral ventricular, cavum septum pellucidum, third ventricular and fourth ventricular volumes. Intracranial volume (ICV) was calculated by summing TTV and CSF volumes. Examples of these segmentations for a fetus in the 22q cohort and the matched control cohort, along with 3-Dimensional volumetric representations are shown in Fig. 1.

### Statistics

All statistical analyses were performed using statsmodels (v0.13.2) [28] and Jupyter Notebook, python3. The Shapiro-Wilk test was used to test normality. A Kruskal-Wallis test was performed to test for group differences in GA at scan, total brain tissue volume and each of the brain tissue regions described above. For any regions where a significant group difference was identified, a post-hoc analysis was undertaken using a Mann-Whitney U-test to assess whether volumes were significantly different between each of the three cohorts. P-values < 0.05 were considered significant. The effect-size of group on TTV and each individual brain tissue region was calculated pair-wise between each of the three cohorts using Cohen’s D, using the Mann-Whitney U-test statistic, to estimate the magnitude of difference between groups, as described previously [29]. Cohen’s D effect sizes were categorised as small (0.2 ≤ d < 0.5), medium (0.5 ≤ d < 0.8), and large (d ≥ 0.8).

## Results

**Fig. 2:**
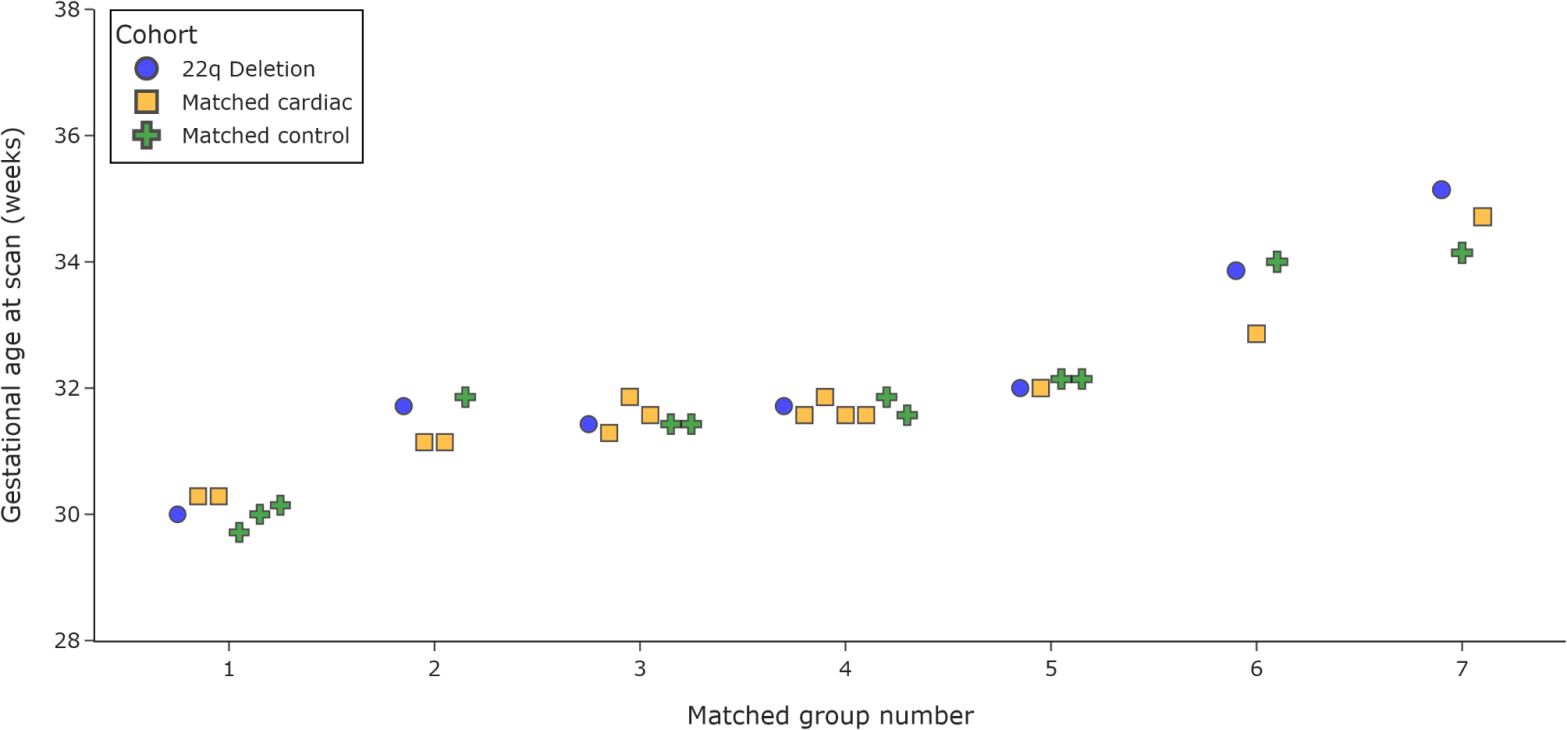
Fetal sex and gestational age at scan for all participants (seven groups, one for each 22qDS participant)

33 fetuses were included in this study: 7 in the 22q cohort, 14 in the matched cardiac cohort and 12 in the matched control cohort (Fig. 2). Full demographics can be found in Table 1. There was no significant difference in GA at the time of the scan between groups (H = 0.67, *p* = 0.72). Radiological reports for all fetuses in the matched cardiac or matched control cohort reported appropriate appearances to the brain for GA. No structural brain or extra-cardiac abnormalities were identified in these two groups.

**Table 1:** Cohort demographics

Cohort	22qDS	Cardiac	Control
**N (Male: Female)**	7 (4:3)	14 (10:4)	12 (8:4)
**Gestational age at scan**,** weeks (median [IQR])**	31.71 [31.57-33.00]	31.71 [31.17–31.86]	31.71 [31.11–32.15]
**Primary cardiac defect**,** N**	Isolated right aortic arch: 2 Common arterial trunk: 2 Interrupted aortic arch: 2 Pulmonary atresia + VSD: 1	Isolated right aortic arch: 8 Common arterial trunk: 4 Interrupted aortic arch: 2 Pulmonary atresia + VSD: 2	-
**Structural brain abnormalities**,** N**	Prominent cavum septum pellucidum: 1	-	-
**Extracardiac abnormalities**,** N**	Absent or hypoplastic thymus: 3	-	-

Demographics of all 33 fetuses included in the study

**Fig. 3:**
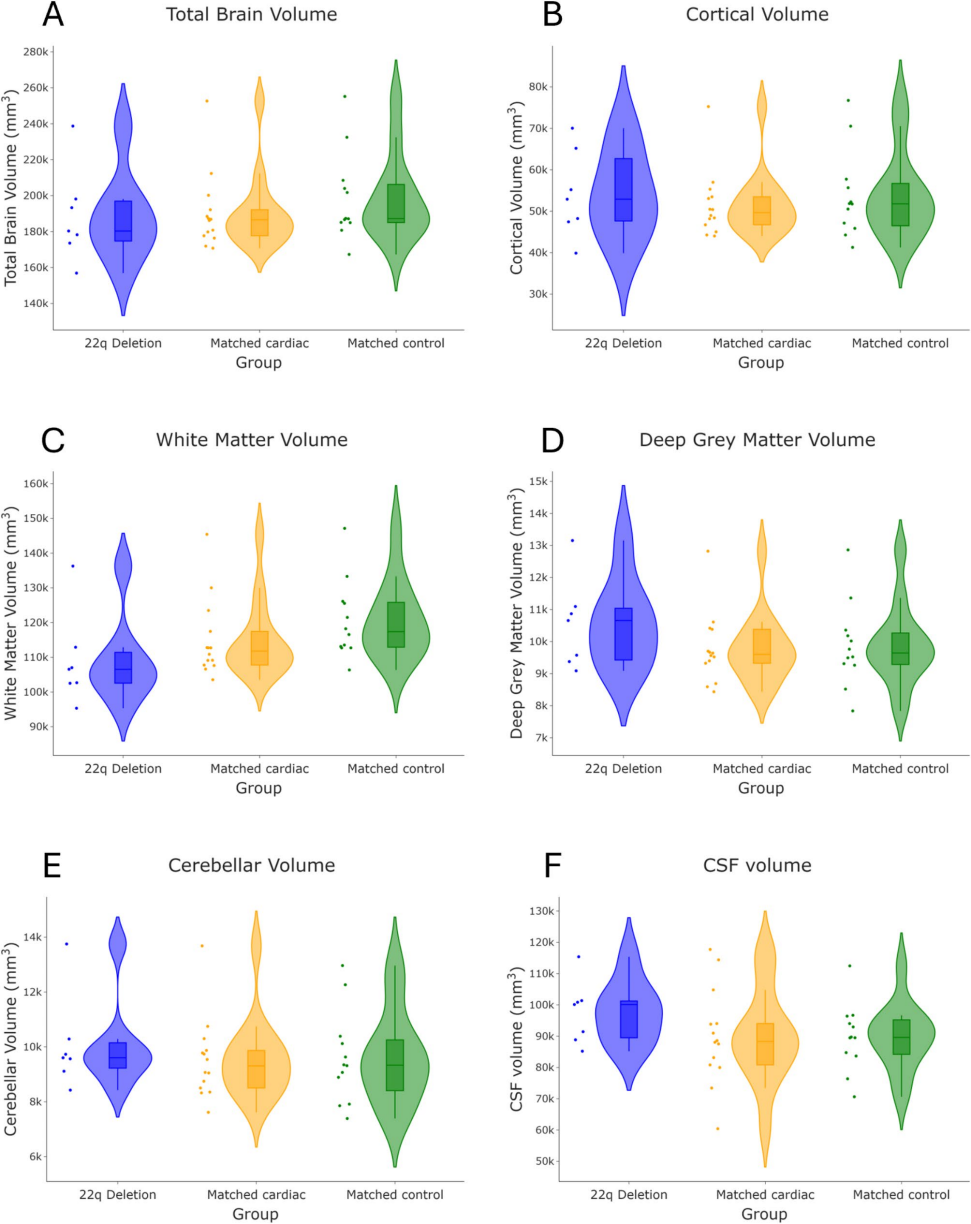
Violin plots showing volumes for six different regions (**A**) Total brain tissue; (**B**) cortical grey matter; (**C**) white matter (**D**) deep grey matter volume; (**E**) Cerebellum; and (**F**) Cerebrospinal Fluid for fetuses with 22q deletion syndrome (blue), matched cardiac fetuses (yellow) and healthy control fetuses (green)

There were no significant group differences in total brain tissue volumes, the volumes of cGM, dGM, the cerebellum, CSF or intracranial volumes between fetuses with 22qDS or sex and gestation-matched cardiac or control fetuses. There was a significant difference in WM volumes between these groups (H = 6.97, *p* = 0.031) (Table 2; Fig. 3). Post-hoc analyses showed that WM volumes were significantly reduced in fetuses with 22qDS when compared to healthy control fetuses (U = 16.0, *p* = 0.028), but there were no significant differences in WM volumes between fetuses with 22qDS and sex and gestation-matched cardiac fetuses (U = 26, *p* = 0.09), or between fetuses in the matched-cardiac and healthy control cohorts (U = 50, *p* = 0.09).

**Table 2:** Total and regional brain volumes for each of the three cohorts

Region	Median (IQR) volume (mm^3^)			*P*-value*
	22qDS cohort (*N* = 7)	Cardiac cohort (*N* = 14)	Control cohort (*N* = 12)	
Total Brain Tissue	180,336 (175,915 − 195,722)	186,532 (178,217 − 191,228)	187,245 (185,085–205,130)	0.43
Cortical grey matter	52,904 (47,848 − 60,194)	49,686 (47,099 − 53,381)	51,802 (46,823 − 56,184)	0.80
White matter	106,555 (102,600 − 109,962)	111,804 (108,100–116,302)	117,391 (113,012–125,685)	**0.031**
Deep grey matter	10,657 (9,476 − 10,982)	9,603 (9,344 − 10,213)	9,644 (9,299 − 10,226)	0.42
Cerebellum	9,604 (9,340 − 10,010)	9,307 (8,565-9,848)	9,333 (8,648 − 10,188)	0.71
Cerebrospinal fluid	100,118 (90,140 − 10,1103)	88,321 (81,403 − 93,980)	89,614 (84,471 − 94,621)	0.26
Intracranial volume	280,454 (262,938 − 304,093)	271,201 (264,824 − 293,489)	277,345 (273,772 − 306,111)	0.85

Total brain tissue volumes and volumes of different intracerebral tissue types for each of the three groups: 22q, matched cardiac and matched control fetuses

* P-value derived from Kruskal-Wallis test. Values in **bold** are considered significant

Additional post-hoc analyses revealed significant group differences in WM volumes as a proportion of total brain tissue volume (Median [IQR] WM: TTV ratio: 22qDS fetuses = 0.58 [0.57–0.59]; Cardiac fetuses = 0.61 [0.59–0.62]; Control fetuses = 0.61 [0.60–0.63]); H = 7.67, *p* = 0.022). WM: TTV ratio was significantly lower in the 22qDS fetuses compared to both the cardiac fetuses (U = 16.0, *p* = 0.012) and the control fetuses (U = 13.0, *p* = 0.013), but there were no significant differences in WM: TTV ratio between the cardiac and control cohorts (U = 73.0, *p* = 0.59). Pair-wise effect-sizes between each of the three cohorts are shown in Table 3. Large effect-sizes were seen between the 22qDS and cardiac cohort (D_Cohen_ = 0.81), and between the 22qDS and control cohort (D_Cohen_ = 1.2) for WM volumes.

**Table 3:** Pairwise effect sizes for total and regional brain volumes between groups

Region	Pair-wise group comparison		
	22qDS vs. cardiac	22qDS vs. control	Cardiac vs. control
**Total Brain Tissue**	U = 46, *p* = 0.86 D_Cohen_ = 0.098	U = 28, *p* = 0.26 D_Cohen_ = 0.56*	U = 64, *p* = 0.32 D_Cohen_ = 0.41
**Cortical grey matter**	U = 55, *p* = 0.69 D_Cohen_ = 0.20	U = 44, *p* = 0.90 D_Cohen_ = 0.078	U = 72, *p* = 0.54 D_Cohen_ = 0.24
**White matter**	U = 26, *p* = 0.09 D_Cohen_ = 0.81^	U = 16, *p* = 0.03 D_Cohen_ = 1.2^	U = 50, *p* = 0.09 D_Cohen_ = 0.73*
**Deep grey matter**	U = 67, *p* = 0.20 D_Cohen_ = 0.61*	U = 54, *p* = 0.34 D_Cohen_ = 0.48	U = 84, *p* = 0.99 D_Cohen_ = 0.00
**Cerebellum**	U = 59, *p* = 0.49 D_Cohen_ = 0.33	U = 51, *p* = 0.48 D_Cohen_ = 0.35	U = 84, *p* = 0.99 D_Cohen_ = 0.00
**Cerebrospinal fluid**	U = 68, *p* = 0.17 D_Cohen_ = 0.65*	U = 60, *p* = 0.14 D_Cohen_ = 0.75*	U = 78, *p* = 0.76 D_Cohen_ = 0.12
**Intracranial volume**	U = 56, *p* = 0.64 D_Cohen_ = 0.23	U = 41, *p* = 0.97 D_Cohen_ = 0.039	U = 66, *p* = 0.37 D_Cohen_ = 0.12

Pairwise comparison performed using Mann-Whitney U-Test. Cohen’s D (D_Cohen_) effect sizes were categorised as small (0.2 ≤ d < 0.5), medium (0.5 ≤ d < 0.8), and large (d ≥ 0.8)

^ indicates large effect sizes between groups. * indicates medium effect sizes between groups

## Discussion

In this first small, exploratory study using MRI to explore brain volumes in a small number of fetuses with 22qDS we show that absolute WM volumes are significantly reduced when compared to gestation and sex-matched healthy fetuses. No significant difference was seen in WM volumes between fetuses with 22qDS and CHD when compared to gestation and sex-matched fetuses with the same CHD diagnosis but without 22q11.2 deletion. However, WM volumes as a proportion of total brain tissue volume were significantly reduced in fetuses with 22qDS and CHD when compared to gestation and sex-matched fetuses with the same CHD diagnoses as those with 22qDS but without 22q11.2 deletion, as well as to the healthy control cohort. There was a trend towards smaller total brain tissue volumes and larger CSF volumes in the 22qDS cohort compared to both the healthy control and matched-cardiac cohort, but these results did not reach statistical significance.

The perspective on multifaceted syndromes, as in 22qDS, continues to shift as improved postnatal management (i.e. corrective surgery in accompanying CHD), has increased life expectancies and thus raised awareness of earlier cognitive impairments and later-stage neuropsychiatric impairments in affected patients. High incidences of schizophrenic disorders in 22qDS are of particular concern when it comes to leading an independent life and call for a thorough investigation into potential neurodevelopmental causes [30]. Concomitant CHD in 22qDS, which is known to be independently associated with impaired brain development [15, 31, 32], could be a significant confounder when comparing this patient cohort to normal fetuses and thus render causative statements on brain dysgenesis difficult. Fetal MRI offers unprecedented qualitative and quantitative insights into in utero brain development, hence shaping our understanding of gestation-dependent brain pathophysiology.

Using data from three independent fetal cohorts, we were able to show that a reduction in total WM volumes and in WM volumes as a proportion of TTV could be a feature of fetal 22qDS brain phenotypes, when compared to typical development. This is aligned with prior observations in the paediatric and adult populations, for whom reductions in white matter volumes as well as structural abnormalities in the cerebral WM have been reported [33, 34]. Identifying this reduction in WM volumes in utero lends support to the theory of an early, potentially genetics-driven, developmental mechanism over secondary brain impairment linked to accompanying CHD. This hypothesis may be supported by the fact that several genes on the 22q locus have been linked to essential steps in WM maturation [35–37] The finding that WM volumes as a proportion of TTV are reduced in fetuses with 22qDS and CHD suggests that reductions in WM volume aren’t simply due to smaller overall brain volumes, but rather to specific reductions in WM volume independent of any impairment in overall brain growth that might occur.

Structural MRI has suggested that cortico-cortical association fibres, the interconnectivity of which plays an eminent role in neuropsychiatric disorders, are particularly reduced in 22qDS [38]. Albeit speculative, a sequence in which 22q-linked gene deletions impair cortico-cortical fibre growth, leading to both reduced WM volumes and impaired cortical interconnectivity thus seems plausible. Future research in this domain could forge a better understanding of these processes and may open the door for designing molecular targets.

The reduction in WM reported here adds further important knowledge on the expected trajectory of brain development in 22qDS. Notably, these changes would probably not have been picked up by conventional postnatal measurements of head circumference as total intracranial volume was similar across groups. Prospectively, even earlier and more detailed insights into brain development could help explain the neuropsychiatric vulnerability in 22qDS patients compared to isolated CHD [39].

Previous reports often describe increased cortical thickness in the paediatric and adult 22qDS population [40–43]. Whilst we have not directly investigated cortical thickness, in our analysis fetal cortical grey matter volumes seemed to be less affected than WM volumes. This finding may be related the small sample size, and to differences in the age at which these measurements are obtained. Pinpointing a monocausal process influencing cortical development is difficult, as many factors influence neocortical development. Cortical thickness tends to peak after infancy [44], and cortical thinning is a physiological process occurring during adolescence and strongly correlated with WM maturation, forbidding straightforward comparisons across age-groups [45].

Abnormal interneuron migration due to CXCR4 gene deficiency on the 22q11.2 locus has nonetheless been recently proposed as a molecular mechanism leading to reduced cortical thickness on 22qDS, given its causative role in interneuron migration [46]. Even though one could argue that the spatial resolution and partial-volume effects associated with fetal MRI makes it challenging to detect subtle and region-specific volumetric changes, our observed discrepancy between WM and GM volumes remains of interest. It may be related to the smaller numbers involved in this study compared to previous work [33, 34]. It is conceivable that WM impairment precedes cortical dysgenesis and may even contribute to it due to abnormally reduced cortico-cortical interconnectivity. Hence, reduced WM volumes might only be the measurable ‘tip-of-the-iceberg’ behind more complex corticomedullary dysgenesis. Alternatively, a two-hit theory of individually affected WM and GM seems to be supported by the genetic deletions specifically targeting essential steps in the development of both compartments.

### Limitations

Even though this is the first fetal brain MRI study in 22qDS, the relatively low numbers included weaken the robustness and statistical power of the effects that we observed. The small sample sizes also make it likely that only large effect sizes would be detected. Due to the small number of fetuses involved and the preliminary nature of this study, we did not correct for multiple comparisons. We also did not explore the effect of isolated 22qDS *without* CHD. This remains difficult to assess as individuals with 22qDS but without any cardiac or extra-cardiac anomalies are not typically identified in utero on routine screening. The range of CHD diagnoses included was broad, including isolated aortic arch anomalies, conotruncal defects and right-sided obstructive cardiac lesions. Some of these diagnoses are not necessarily associated with a reduction in cerebral substrate delivery or brain volumes [15, 31, 47] and may explain why no significant differences in total or regional brain volumes between the control and cardiac cohorts were identified. Furthermore, low ratios of absolute tissue volumes to voxel size for some regions explored may make it challenging to detect subtle volumetric changes at early GAs.

Future, larger studies are required, including individuals with 22qDS but no concomitant CHD where possible. Future work performing comparable analyses at the neonatal time-point would be valuable, with the additional benefit that more complex imaging may be possible. MRI techniques such as diffusion MRI imaging have proved a valuable way of assessing WM development in paediatric and adult populations and could potentially add microstructural information beyond the volumetric changes observed here. Multidirectional diffusion MRI, however, requires long acquisition times and is therefore more susceptible to motion, making it a technically challenging technique to apply reliably during the fetal period. Acquiring fetal brain images at higher field strengths (i.e. 3 Tesla) in future studies may provide potential benefits of improved signal-to-noise ratio and image resolution.

## Conclusion

In conclusion, we provide the first insights into MRI-assessed fetal brain growth in 22qDS, showing that altered brain development begins in utero in this population. We highlight reductions in WM volumes in fetuses with both 22qDS and CHD when compared to gestation and sex-matched healthy control fetuses, but not to those with the same CHD diagnoses but without 22q11.2 deletion. This suggests that impairments in fetal brain growth in fetuses with 22qDS and concomitant CHD are related to more than just their underlying CHD. A deeper knowledge of in utero brain development may help better understand 22qDS-linked neuropsychiatric disorders and potentially direct research into new therapeutic targets in the future.

## Data Availability

The anonymised numerical datasets analysed during the current study are available from the corresponding author on reasonable request.

## Abbreviations

22qDS: 22q11.2 Deletion Syndrome
CHD: Congenital Heart Disease
MRI: Magnetic Resonance Imaging
cGM: Cortical Grey Matter
WM: White Matter
dGM: Deep Grey Matter
CSF: Cerebrospinal Fluid

## References

[1] McDonald-McGinn DM, Zackai EH, Low D. What’s in a name? The 22q11.2 deletion. Am J Med Genet. 1997;72:247–9. 9382154

[2] Grati FR, Molina Gomes D, Ferreira JCPB, Dupont C, Alesi V, Gouas L, et al. Prevalence of recurrent pathogenic microdeletions and microduplications in over 9500 pregnancies. Prenat Diagn. 2015;35:801–9. 25962607 10.1002/pd.4613

[3] Tan TY, Collins A, James PA, McGillivray G, Stark Z, Gordon CT, et al. Phenotypic variability of distal 22q11.2 copy number abnormalities. Am J Med Genet A. 2011;155A:1623–33. 21671380 10.1002/ajmg.a.34051

[4] Rozas MF, Benavides F, León L, Repetto GM. Association between phenotype and deletion size in 22q11.2 microdeletion syndrome: systematic review and meta-analysis. Orphanet J Rare Dis. 2019;14:195. 31399107 10.1186/s13023-019-1170-x PMC6688301

[5] Gur RE, Yi JJ, McDonald-McGinn DM, Tang SX, Calkins ME, Whinna D, et al. Neurocognitive development in 22q11.2 deletion syndrome: comparison with youth having developmental delay and medical comorbidities. Mol Psychiatry. 2014;19:1205–11. 24445907 10.1038/mp.2013.189 PMC4450860

[6] Swillen A, Moss E, Duijff S. Neurodevelopmental outcome in 22q11.2 deletion syndrome and management. Am J Med Genet A. 2018;176:2160–6. 29696780 10.1002/ajmg.a.38709 PMC6202262

[7] Eliez S, Schmitt JE, White CD, Reiss AL. Children and adolescents with velocardiofacial syndrome: a volumetric MRI study. Am J Psychiatry. 2000;157:409–15. 10698817 10.1176/appi.ajp.157.3.409

[8] Campbell LE, Daly E, Toal F, Stevens A, Azuma R, Catani M, et al. Brain and behaviour in children with 22q11.2 deletion syndrome: a volumetric and voxel-based morphometry MRI study. Brain J Neurol. 2006;129:1218–28. 10.1093/brain/awl066 16569671

[9] Gudbrandsen M, Daly E, Murphy CM, Blackmore CE, Rogdaki M, Mann C, et al. Brain morphometry in 22q11.2 deletion syndrome: an exploration of differences in cortical thickness, surface area, and their contribution to cortical volume. Sci Rep. 2020;10:18845. 33139857 10.1038/s41598-020-75811-1 PMC7606591

[10] Gudbrandsen M, Mann C, Bletsch A, Daly E, Murphy CM, Stoencheva V, et al. Patterns of Cortical Folding Associated with autistic symptoms in carriers and noncarriers of the 22q11.2 Microdeletion. Cereb Cortex N Y N 1991. 2020;30:5281–92. 10.1093/cercor/bhaa108 PMC7566689 32420595

[11] Seitz-Holland J, Lyons M, Kushan L, Lin A, Villalon-Reina JE, Cho KIK et al. Opposing white matter microstructure abnormalities in 22q11.2 deletion and duplication carriers. Transl Psychiatry [Internet]. 2021 [cited 2024 Mar 25];11:1–11. Available from: https://www.nature.com/articles/s41398-021-01703-1 10.1038/s41398-021-01703-1 PMC8581007 34759270

[12] Licht DJ, Shera DM, Clancy RR, Wernovsky G, Montenegro LM, Nicolson SC, et al. Brain maturation is delayed in infants with complex congenital heart defects. J Thorac Cardiovasc Surg. 2009;137:529–36. discussion 536–537. 19258059 10.1016/j.jtcvs.2008.10.025 PMC2701902

[13] Kelly CJ, Makropoulos A, Cordero-Grande L, Hutter J, Price A, Hughes E et al. Impaired development of the cerebral cortex in infants with congenital heart disease is correlated to reduced cerebral oxygen delivery. Sci Rep [Internet]. 2017 [cited 2021 Sep 21];7:15088. Available from: http://www.nature.com/articles/s41598-017-14939-z 10.1038/s41598-017-14939-z PMC5678433 29118365

[14] Kelly CJ, Christiaens D, Batalle D, Makropoulos A, Cordero-Grande L, Steinweg JK et al. Abnormal Microstructural Development of the Cerebral Cortex in Neonates With Congenital Heart Disease Is Associated With Impaired Cerebral Oxygen Delivery. J Am Heart Assoc Cardiovasc Cerebrovasc Dis [Internet]. 2019 [cited 2021 Apr 13];8. Available from: https://www.ncbi.nlm.nih.gov/pmc/articles/PMC6474935/10.1161/JAHA.118.009893 PMC6474935 30821171

[15] Cromb D, Uus A, Van Poppel MPM, Steinweg JK, Bonthrone AF, Maggioni A et al. Total and Regional Brain volumes in fetuses with congenital heart disease. J Magn Reson Imaging JMRI. 2023. 10.1002/jmri.29078 PMC7616254 37846811

[16] Racedo SE, McDonald-McGinn DM, Chung JH, Goldmuntz E, Zackai E, Emanuel BS, et al. Mouse and human CRKL is dosage sensitive for cardiac outflow tract formation. Am J Hum Genet. 2015;96:235–44. 25658046 10.1016/j.ajhg.2014.12.025 PMC4320261

[17] Xu Y-J, Chen S, Zhang J, Fang S-H, Guo Q-Q, Wang J, et al. Novel TBX1 loss-of-function mutation causes isolated conotruncal heart defects in Chinese patients without 22q11.2 deletion. BMC Med Genet. 2014;15:78. 24998776 10.1186/1471-2350-15-78 PMC4099205

[18] Limperopoulos T, Wayne, McElhinney Doff B, Newburger Jane W, Brown David W, Robertson Richard L et al. Brain Volume and Metabolism in Fetuses With Congenital Heart Disease. Circulation [Internet]. 2010 [cited 2020 Oct 13];121:26–33. Available from: https://www.ahajournals.org/doi/10.1161/circulationaha.109.865568 10.1161/CIRCULATIONAHA.109.865568 PMC2819908 20026783

[19] Clouchoux C, Guizard N, Evans AC, du Plessis AJ, Limperopoulos C. Normative fetal brain growth by quantitative in vivo magnetic resonance imaging. Am J Obstet Gynecol. 2012;206:e1731–8. 10.1016/j.ajog.2011.10.002 PMC13054723 22055336

[20] von Rhein M, Buchmann A, Hagmann C, Huber R, Klaver P, Knirsch W, et al. Brain volumes predict neurodevelopment in adolescents after surgery for congenital heart disease. Brain J Neurol. 2014;137:268–76. 10.1093/brain/awt322 24277720

[21] Bulas D, Egloff A. Benefits and risks of MRI in pregnancy. Semin Perinatol. 2013;37:301–4. 24176150 10.1053/j.semperi.2013.06.005

[22] Chartier AL, Bouvier MJ, McPherson DR, Stepenosky JE, Taysom DA, Marks RM. The safety of maternal and fetal MRI at 3 T. AJR Am J Roentgenol. 2019;213:1170–3. 31310182 10.2214/AJR.19.21400

[23] Kuklisova-Murgasova M, Quaghebeur G, Rutherford MA, Hajnal JV, Schnabel JA. Reconstruction of fetal brain MRI with intensity matching and complete outlier removal. Med Image Anal [Internet]. 2012 [cited 2021 Jun 15];16:1550–64. Available from: https://www.ncbi.nlm.nih.gov/pmc/articles/PMC4067058/10.1016/j.media.2012.07.004 PMC4067058 22939612

[24] Uus A, Grigorescu I, van Poppel M, Hughes E, Steinweg J, Roberts T et al. 3D UNet with GAN discriminator for robust localisation of the fetal brain and trunk in MRI with partial coverage of the fetal body [Internet]. Bioengineering; 2021 Jun. Available from: 10.1101/2021.06.23.449574

[25] Uus AU, Egloff Collado A, Roberts TA, Hajnal JV, Rutherford MA, Deprez M. Retrospective motion correction in foetal MRI for clinical applications: existing methods, applications and integration into clinical practice. Br J Radiol [Internet]. 2023 [cited 2023 Sep 22];96:20220071. Available from: https://www.birpublications.org/doi/full/10.1259/bjr.20220071 10.1259/bjr.20220071 PMC7614695 35834425

[26] Uus AU, Kyriakopoulou V, Makropoulos A, Fukami-Gartner A, Cromb D, Davidson A et al. BOUNTI: Brain vOlumetry and aUtomated parcellatioN for 3D feTal MRI. eLife [Internet]. 2023 [cited 2024 Nov 4];12. Available from: https://elifesciences.org/reviewed-preprints/88818

[27] Jenkinson M, Beckmann CF, Behrens TEJ, Woolrich MW, Smith SM. FSL NeuroImage. 2012;62:782–90. 21979382 10.1016/j.neuroimage.2011.09.015

[28] Seabold S, Perktold J, Statsmodels. Econometric and Statistical Modeling with Python. Proc 9th Python Sci Conf. 2010;2010.

[29] Lenhard W, Lenhard A. Computation of Effect Sizes [Internet]. Comput. Eff. Sizes. 2017 [cited 2024 Nov 27]. Available from: http://www.psychometrica.de/effect_size.html

[30] Bassett AS, Chow EWC. Schizophrenia and 22q11.2 deletion syndrome. Curr Psychiatry Rep. 2008;10:148–57. 18474208 10.1007/s11920-008-0026-1 PMC3129332

[31] Peyvandi S, Latal B, Miller SP, McQuillen PS. The neonatal brain in critical congenital heart disease: insights and future directions. NeuroImage. 2019;185:776–82. 29787864 10.1016/j.neuroimage.2018.05.045

[32] Bonthrone AF, Kelly CJ, Ng IHX, Counsell SJ. MRI studies of brain size and growth in individuals with congenital heart disease. Transl Pediatr [Internet]. 2021 [cited 2022 Apr 12];10:2171181–2181. Available from: https://tp.amegroups.com/article/view/56444 10.21037/tp-20-282 PMC8429874 34584889

[33] Kikinis Z, Cho KIK, Coman IL, Radoeva PD, Bouix S, Tang Y, et al. Abnormalities in brain white matter in adolescents with 22q11.2 deletion syndrome and psychotic symptoms. Brain Imaging Behav. 2017;11:1353–64. 27730479 10.1007/s11682-016-9602-x PMC5388603

[34] Villalón-Reina JE, Martínez K, Qu X, Ching CRK, Nir TM, Kothapalli D, et al. Altered white matter microstructure in 22q11.2 deletion syndrome: a multisite diffusion tensor imaging study. Mol Psychiatry. 2020;25:2818–31. 31358905 10.1038/s41380-019-0450-0 PMC6986984

[35] Budel S, Padukkavidana T, Liu BP, Feng Z, Hu F, Johnson S, et al. Genetic variants of Nogo-66 receptor with possible association to schizophrenia block myelin inhibition of axon growth. J Neurosci off J Soc Neurosci. 2008;28:13161–72. 10.1523/JNEUROSCI.3828-08.2008 PMC2892845 19052207

[36] Wang C, Aleksic B, Ozaki N. Glia-related genes and their contribution to schizophrenia. Psychiatry Clin Neurosci. 2015;69:448–61. 25759284 10.1111/pcn.12290

[37] Verdura E, Rodríguez-Palmero A, Vélez-Santamaria V, Planas-Serra L, de la Calle I, Raspall-Chaure M, et al. Biallelic PI4KA variants cause a novel neurodevelopmental syndrome with hypomyelinating leukodystrophy. Brain J Neurol. 2021;144:2659–69. 10.1093/brain/awab124 PMC8557332 34415322

[38] Phillips OR, Nuechterlein KH, Asarnow RF, Clark KA, Cabeen R, Yang Y, et al. Mapping corticocortical structural integrity in schizophrenia and effects of genetic liability. Biol Psychiatry. 2011;70:680–9. 21571255 10.1016/j.biopsych.2011.03.039 PMC3838300

[39] Yi JJ, Tang SX, McDonald-McGinn DM, Calkins ME, Whinna DA, Souders MC, et al. Contribution of congenital heart disease to neuropsychiatric outcome in school-age children with 22q11.2 deletion syndrome. Am J Med Genet Part B Neuropsychiatr Genet off Publ Int Soc Psychiatr Genet. 2014;165B:137–47. 10.1002/ajmg.b.32215 PMC4154196 24265253

[40] Bagautdinova J, Zöller D, Schaer M, Padula MC, Mancini V, Schneider M et al. Altered cortical thickness development in 22q11.2 deletion syndrome and association with psychotic symptoms. Mol Psychiatry [Internet]. 2021 [cited 2024 Nov 27];26:7671–8. Available from: https://www.ncbi.nlm.nih.gov/pmc/articles/PMC8873018/10.1038/s41380-021-01209-8 PMC8873018 34253864

[41] Schmitt JE, Vandekar S, Yi J, Calkins ME, Ruparel K, Roalf DR, et al. Aberrant cortical morphometry in the 22q11.2 deletion syndrome. Biol Psychiatry. 2015;78:135–43. 25555483 10.1016/j.biopsych.2014.10.025 PMC4446247

[42] Sun D, Ching CRK, Lin A, Forsyth JK, Kushan L, Vajdi A, et al. Large-scale mapping of cortical alterations in 22q11.2 deletion syndrome: convergence with idiopathic psychosis and effects of deletion size. Mol Psychiatry. 2020;25:1822–34. 29895892 10.1038/s41380-018-0078-5 PMC6292748

[43] Jalbrzikowski M, Lin A, Vajdi A, Grigoryan V, Kushan L, Ching CRK et al. Longitudinal trajectories of cortical development in 22q11.2 copy number variants and typically developing controls. Mol Psychiatry [Internet]. 2022 [cited 2024 Nov 27];27:4181–90. Available from: https://www.nature.com/articles/s41380-022-01681-w 10.1038/s41380-022-01681-w PMC9718681 35896619

[44] Bethlehem Ra, Seidlitz I, White J, Vogel SR, Anderson JW, Adamson KM. Brain charts for the human lifespan. Nature. 2022;604:525–33. 35388223 10.1038/s41586-022-04554-y PMC9021021

[45] Jeon T, Mishra V, Ouyang M, Chen M, Huang H. Synchronous changes of cortical thickness and corresponding White Matter Microstructure during Brain Development accessed by Diffusion MRI Tractography from Parcellated Cortex. Front Neuroanat. 2015;9:158. 26696839 10.3389/fnana.2015.00158 PMC4667005

[46] Stumm RK, Zhou C, Ara T, Lazarini F, Dubois-Dalcq M, Nagasawa T, et al. CXCR4 regulates interneuron migration in the developing neocortex. J Neurosci off J Soc Neurosci. 2003;23:5123–30. 10.1523/JNEUROSCI.23-12-05123.2003 PMC6741192 12832536

[47] Vigneswaran T, Zidere V. Aortic Arch Abnormalities. In: Simpson J, Zidere V, Miller OI, editors. Fetal Cardiol Pract Approach Diagn Manag [Internet]. Cham: Springer International Publishing; 2018 [cited 2023 Sep 12]. pp. 139–51. Available from: 10.1007/978-3-319-77461-9_9

